# Caveolin-1 regulates OMV-induced macrophage pro-inflammatory activation and multiple Toll-like receptors

**DOI:** 10.3389/fimmu.2023.1044834

**Published:** 2023-02-02

**Authors:** Ayyanar Sivanantham, Ward Alktaish, Selvakumar Murugeasan, Bin Gong, Heedoo Lee, Yang Jin

**Affiliations:** ^1^ Division of Pulmonary and Critical Care Medicine, Department of Medicine, Boston University, Boston, MA, United States; ^2^ Department of Chemical Engineering, Indian Institute of Technology, Tirupati, Andhra Pradesh, India; ^3^ Department of Pathology, University of Texas Medical Branch, Galveston, TX, United States; ^4^ Department of Biology and Chemistry, Changwon National University, Changwon, Republic of Korea

**Keywords:** macrophage activation, inflammation, bacterial infection, OMV, bacteria, caveolin-1, TLRs

## Abstract

Macrophages (MФ), the primary cell of the innate immune system, serves as the first line of defense. During bacterial infection, Gram-negative (G-) bacteria release nanosized outer membrane vesicles (OMVs), facilitating the crosstalk between the microbe and the host. The underlying mechanisms by which OMVs induced pro-inflammatory (M1) activation are still unknown. Our study shows that OMVs caused M1 activation *via* modulating various toll-like receptor (TLR) expressions as they contain LPS, LTA, bacterial DNAs, and flagellins. Also, we found that caveolin-1 (cav-1), a 21-kDa scaffolding protein of caveolae and lipid rafts, plays a significant role in OMV-induced pro-inflammatory response in regulating various TLR signaling pathways. Specifically, cav-1 deletion increased the expression of OMV-induced TLRs, pro-inflammatory cytokine secretions (TNF-α and IL-1β), and the reactive oxygen species (ROS) production in MФs. Further, we examined the interaction between Cav-1 and TLR4 by immunoprecipitation, colocalization, and computational models, providing future direction to explore the role of cav-1 in OMV-induced other TLR signaling. Altogether, Cav-1 is a key regulator in OMV-induced multiple TLRs response. This study promotes future research to develop drugs by targeting the specific motif of cav-1 or TLRs against bacterial infection and macrophage-mediated inflammation.

## Introduction

Macrophages (MФ) are eventually present in all organs and tissues, constituting the first line of defense in the host immune system. They have functional diversity with many receptors to recognize antigens and bind to the Fc region of immunoglobulin G, complement fragments, and mannose residues in bacteria (1–3) In response to the pathogenic invasion, MФ can acquire distinct phenotypic polarization, referred to as M1- (classically activated) MФ that release pro-inflammatory mediators, along with nitric oxide and reactive oxygen species (ROS) required for host cell defense and the removal of intracellular pathogens. However, extensive M1 MФ polarization can cause severe damage to the host (4, 5). MФ recognizes the pathogenic bacteria *via* pattern recognition receptors (PRRs) of toll-like receptors (TLRs) by interacting with microbe-associated molecular patterns (MAMPs). It mediates the bacterial-associated M1 polarization and innate immune responses (6–9).

TLRs, type I transmembrane glycoproteins, contains a leucine-rich repeat (LRR) motif, the transmembrane helix, and the cytoplasmic domain (10, 11). Extracellular LRR acts as a ligand-binding domain, whereas the cytoplasmic domain serves as a platform for downstream cell signaling for TLRs. According to the subcellular location, ten functional TLRs have been identified in humans (TLRs 1–10) (10), whereas, in mice, it’s twelve (TLRs 1–9 and 11–13) (12). Among those, TLR1, TLR2, TLR4, TLR5, TLR6, and TLR10 are present on the cell surface, TLR3, TLR7, TLR8, and TLR9 are expressed in internal compartments, especially in endosomes, and the endoplasmic reticulum. Currently, well-known ligands for TLRs include Triacylated lipopeptides (TLR1-2), lipoteichoic acid and peptidoglycan (TLR2), dsDNA (TLR3), LPS (TLR4), ssRNA (TLR7-8), Flagellin (TLR5), and profilin (TLR11). During pathogenesis, various combinations of TLRs were found to express in different subsets of immune and non-immune cell types, such as monocytes, macrophages, dendritic cells, neutrophils, and lymphocytes (6, 7, 11).

Outer membrane vesicles (OMVs) are bilayered spherical particles released into the extracellular space by the gram-negative (G-) bacteria in the size of 20 to 400 nm (13). It contains outer membrane proteins (OMPs), lipopolysaccharides (LPS), peptidoglycan, phospholipids, cell wall components, periplasmic and cytoplasmic proteins, nucleic acids, and ion metabolites. Although the OMVs were first found to be reported in G- bacteria, emerging studies indicate that gram-positive (G+) bacteria also release OMVs under a different mechanism (14–16). OMV mediates the bacteria-bacteria and bacteria-host interactions. Multiple OMV components play vital roles in MФ polarization and activation *via* direct uptake or triggering a signaling pathway of the host cell surface antigens (16). Further reports showed that OMVs play an essential role in inducing macrophage apoptosis, pyroptosis, and NLRP3 inflammasome activation, as well as the production of pro-inflammatory mediators, thereby exacerbating the inflammatory responses (17–20). A recent study shows that OMVs derived from *Acinetobacter baumannii* (*A. baumannii*) promote the secretion of inflammatory mediators through TLR2- and TLR4 pathways using the murine model (21).

Multiple TLRs can interact with components of the plasma membrane, such as Caveolin-1 (Cav-1), to regulate phagocytosis and cell activation (22–25). Cav-1, a membrane scaffolding protein (22 to 25 kDa) present in caveolae and lipid rafts, exerts several functions, including metabolism, endocytosis, exocytosis, and signal transduction (22, 26, 27). The hairpin-like structure of cav-1 holds four key structural domains: the N-terminal domain (residues 1–81), the scaffolding domain (residues 82–101), the intramembrane domain (residues 102–134), and the C-terminal domain (residues 135–178). The N- and C-terminals of cav-1 were separated by an intramembranous hydrophobic loop that faces the cytoplasm (27). Some studies reported that cav-1 involves in inflammatory responses by regulating TLR2, 4, and 5 (28–30). Nevertheless, cav-1 functions may differ depending on the pathogen and host cell types (22, 31, 32).

However, the relationship between Cav-1 and G- bacterial OMV-induced TLRs expression needs to be better understood. Therefore, this brief report aims to illustrate the effect of OMVs on MФ activation and different TLRs in the presence or absence of cav-1. Our study evaluates the relationship of cav-1 with TLRs and provides a novel insight into the future of OMV-related research.

## Methods

### Bacterial culture maintenance

*E. coli* O6: K2:H1 (ATCC, Manassas, VA.) was pre-cultured in Luria-Bertani (LB) medium under overnight shaking at 37°C. Following this, a 1:1000 dilution of the pre-culture was added to a fresh LB medium and incubated until 12h. Bacterial growth was measured at OD600 using SmartspecTM Biorad. The bacterial concentration was calculated using the formula OD600 = 8 x 10^8^ cells/ml with a value of 1.0.

### Isolation and characterization of OMV

30 ml of E. coli in the late log phase were centrifuged twice for 30 min at 5000 x g and 4°C. The cell-free supernatant was filtered using pore sizes of 0.45 and 0.22μm filters. OMVs were isolated from the filtered supernatant using the ExoBacteria OMV isolation kit (System Biosciences, Palo Alto, CA), followed by the manufacturer’s instructions. Collected OMVs were purified with sterile PBS (three times) using Amicon^®^ Ultra-15 10,000 Da filters at 10,000xg for 10min and 4°C. Then, the residual OMVs were dissolved in sterile PBS, and the Total protein content of OMVs was determined by the Bradford method.

Transmission electron microscopy (TEM) analysis, 5µl OMVs was adsorbed for 1 minute to a carbon-coated grid that had been made hydrophilic by a 20sec exposure to a glow discharge (25mA); the grid was then floated briefly on a drop of water, then stained with 0.75% uranyl formate for 15 seconds. After removing the excess uranyl formate, the grids were examined in a JEOL 1200EX TEM, and images were recorded with an AMT 2k CCD camera.

General Lipopolysaccharides (LPS) ELISA Kit (MyBioSource) was used to measure the LPS presence in 100µl of OMVs, followed by the manufacturer’s instructions.

### Animals

Six to eight weeks old male C57BL/6 Wild type (WT) and cav-1 KO mice (strain #007083) were supplied by Jackson Laboratory (Bar Harbor, ME) and were used at the age of 8 weeks. All the mice were kept in a specific pathogen-free animal facility at the Boston University School of Medicine. All animal-related procedures were approved and followed the guidelines by Boston University’s institutional animal care and use committee (IACUC) (# PROTO201800354/PROTO201800355).

### Isolation and differentiation of alveolar MФs (AM)

Animals were euthanized, and the BALF was collected using BAL buffer containing 1X PBS with 2 mM EDTA and 0.5% FBS. Collected BALF was filtered through a 70μm cell strainer into the 15-ml tube containing 3 ml of DMEM with 10% FBS. Then, cells were pelleted from BAL fluid by centrifugation for 300xg, 5min at 4°C. RBCs were lysed using 1X RBC lysis buffer. The macrophages were cultured in DMEM supplemented with 20% L-929 conditioned medium (as a source of M-CSF), 10% FBS, 1 mM Sodium Pyruvate, 10 mM HEPES, and 1x antibiotics for at least 5-7 days to permit differentiation and used for further experimentation (33).

### Isolation of bone marrow-derived MФs (BMDM)

Mouse femurs and tibias were flushed with 1XPBS and filtered through a 70μm cell strainer. The macrophage-containing flow-through was cultured with DMEM supplemented with 30% L-929 conditioned medium (as a source of M-CSF), 10% FBS, 1 mM Sodium Pyruvate, 10 mM HEPES, and 1% antibiotics for 5-7 days to allow for differentiation and used for further experiments (34, 35).

### THP-1 MФ culture and transfection of Cav-1 siRNA

Human THP1 monocytes were obtained from the American Type Culture Collection (ATCC) and maintained in RPMI-1640 with 10% fetal bovine serum (FBS) and 1% penicillin/streptomycin. Cells were treated with 20 ng/ml Phorbol 12-myristate 13-acetate (PMA) (Sigma-Aldrich), kept for 72 h to differentiate into macrophages, then matured for another 24h in a fresh medium. Cells were then transfected with 25µM of Cav-1 siRNA and a negative control using jetPRIME transfection reagent as per the manufacturer’s instruction.

All the macrophages were incubated at 37°C in a humidified incubator with 5% CO_2_.

### OMV or E. coli treatment

Cells were treated with 100µl of Elution buffer/PBS (used to elute OMV), or two different doses of OMV or 3.68X10^6^ fixed E. coli (used to get 0.25µg/ml) after overnight serum starvation. The sample was then collected for subsequent experiments at the appropriate time points.

### Reactive oxygen species (ROS), MФ migration, and phagocytosis

ROS expression in the cells treated with EB or OMV was measured using the CM-H2DCFDA assay method (Invitrogen). Briefly, after 24h of EB or OMV treatment, cells were subjected to 10µM of CM-H2DCFDA containing dye and incubated for 1h under 37°C with 5% CO2; Excitation and emission were observed with 492 and 520 nm (using Promega Glomax explorer), respectively. The observed fluorescence unit (FU) was directly proportional to the ROS production in the cells.

MФ migration assay, Cells were cultured in the inner chamber containing inserts with a pore size of 8.0 mm and a diameter of 6.5 mm. Meanwhile, 10%FBS-containing medium with or without OMV was added to the outer well and incubated for 24h. Using a light microscope, migrated cells were counted after fixation and H&E staining.

100μg/ml of pHrodo™ Green E. coli BioParticles™ Conjugate (Thermofisher Scientific, Waltham, MA) dissolved in complete media and introduced to macrophages treated with EB or OMV incubated for 1h under 37°C. Then, fluorescence excitation and emission were observed with 509 and 533 nm, respectively.

### Inflammatory cytokines analysis

Inflammatory cytokines gene expression and their secretion were analyzed. Macrophages were lysed, and total RNA was isolated by the TRIzol (Invitrogen) method. Then, 1µg of total RNA was reverse transcribed using a high-capacity cDNA reverse transcription kit. The expression levels of various genes (primer sequence **Supplementary Table 1**) were quantified using SYBR green and a detection system. The relative expression for each gene was normalized with GAPDH.

TNF-α and IL-1β ELISA kits (R&D systems) were used to measure the cytokine concentration in the cell-free supernatant as per instructions provided by the manufacturer.

### Immunoblotting analysis

After 3h of OMV treatment, cells were lysed using Radioimmunoprecipitation assay (RIPA) Lysis and Extraction Buffer, and protein quantification was done by the Bradford method. Equal quantities of protein were separated by 10% SDS-PAGE and transferred to a polyvinylidene fluoride (PVDF) membrane probed with specific primary antibodies overnight at 4°C and incubated with the appropriate secondary antibodies. Enhanced chemiluminescence (ECL) detection reagent was used for the band visualization. GAPDH was employed as an internal protein control. Bands were quantified based on the densitometry method using ImageJ software.

### Microscopic analysis

TLRs and cav-1 expression were analyzed using a fluorescence microscope, and colocalization of TLR4 and Cav-1 was investigated by the confocal microscopic method.

Briefly, Cells were seeded into Lab Tek II Chamber Slides (fluorescence) or 35mm glass bottom dish (for confocal); after cell attachment, they starved overnight. Then, cells were treated with OMVs for 3h. Next, cells were fixed in ice-cold methanol for 20min under -20°C and permeabilized with permeabilizing buffer (PBS + 0.2% Tween20) for 5min. Cells were blocked using 1% BSA for 30min. The blocked cells were probed with specific primary antibodies (1:100) overnight at 4°C and FITC and Alexa Flour 594 conjugated secondary antibodies (1:100) for 1h at room temperature. Cells were washed three times within PBS and mounted with slowFade™ Diamond Antifade mountant with DAPI. Fluorescence was observed under LEICA DM4B, and colocalization was observed under Zeiss LSM 700 Laser Scanning Confocal Microscope and photographed. ImageJ was used to measure the fluorescent intensity and colocalization coefficient.

### Co-immunoprecipitation (CO-IP)

Cells were lysed with IP-lysis buffer (Thermo Scientific), and protein concentration was measured with the Bradford method. 100µg of protein was incubated with 5µg of Cav-1 antibody and gently rotated at 4°C overnight. The immunocomplex was collected using Pierce™ Crosslink Magnetic IP/Co-IP Kit per the manufacturer’s instructions. The immunoprecipitated protein was solubilized with a sample buffer and analyzed through immunoblotting with an anti-TLR4 antibody. Meanwhile, 20% of the cell lysate is used as input.

### Cav-1 and TLRs interaction prediction

Protein sequences and 3D structures of Cav-1 and TLR4 were obtained from UniPort and Protein Data Bank database (Cav-1: Q03135 & TLR-4: 3FXI). The protein structures Cav-1 with TLR4 were subjected to interaction studies using the pyDockWEB. The obtained docked complexes were visualized by UCSF Chimera molecular visualization program.

### Statistical analysis

Results were stated as mean ± SD. Non-parametrical statistical analysis was performed by a One-way Analysis of variance (ANOVA) test with Tukey’s multiple comparisons to determine significant differences between the experimental groups.

## Results

### Cav-1 deletion enhances OMVs-induced MФ polarization by increasing ROS production and cytokine secretion

**Figure 1:**
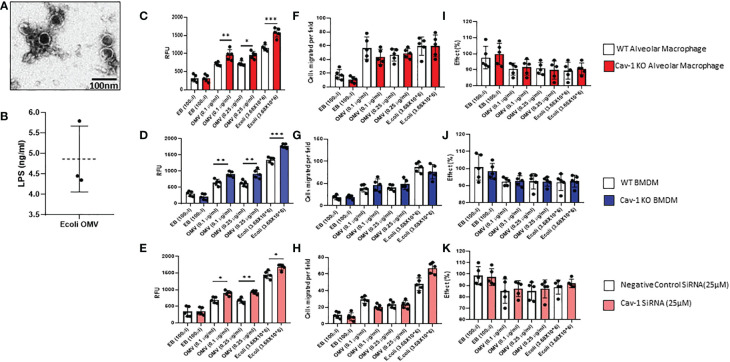
Effect of OMVs on WT and Cav-1 KO AMФ, BMDM, and human THP-1 cells. **(A)** Representative Transmission electron microscopy (TEM) image of OMVs isolated from *E. coli* (scale bar = 100nm). **(B)** Graphical representation of LPS presence in *E.coli* OMVs was quantified using a competitive inhibition ELISA method (n=3). Cells were treated with different concentrations (0.1 and 0.25μg/ml) of OMVs or *E. coli* (3.68X10^6^); after 24h, OMV-induced ROS secretion in all the macrophages using the CM-H2DCFDA assay method **(C–E)**. Migration assay of OMV-treated AM, BMDM, and THP-1 cells. Cells were treated with OMVs (0.10 or 0.25μg/ml), and their migration was assessed **(F–H)**. Effect of OMV on Phagocytosis in macrophages **(I–K)** (n = 5). Results were expressed as mean ± SD. Statistical Analysis was performed non-parametrically using the One-way Analysis of variance (ANOVA) with Tukey’s multiple comparison tests to determine significant differences between the experimental groups. *p < 0.05, **p<0.01 and ***p<0.005 set as Statistical significance.

In TEM analysis, OMVs were surrounded by a bilayer membrane with diameters below 100nm and thus appeared as spherical structures (**Figure 1A**). By measuring the total LPS in the OMVs, it consists of approximately 5 ng/ml of LPS (**Figure 1B**). To study the effects of OMVs on MФ activations and examine the role of cav-1, we treated macrophages with OMVs in a dose-dependent manner. **Figure 1** shows two primary mouse MФ, including the primary alveolar macrophages, BMDMs, and one human macrophage cell line (THP-1cells), were used. Elution buffer (EB) was used as the negative control and fixed *E. coli* was used as the positive control. 0.1 µg/ml and 0.25 µg/ml of OMV treatment significantly increased ROS production (**Figures 1C–E**) and cell migration (**Figures 1F–H** & **Supplementary Figure 1E**) and did not make a significant impact on the phagocytosis (**Figures 1I–K**) on all three MФ. Deletion of cav-1 significantly augmented the effects of OMVs on MФ-derived ROS production in all three types of MФ. Meanwhile, cav-1 deletion does not significantly alter cell migration and phagocytosis, suggesting that cav-1 may not involve in these two activities (**Figures 1C–K**). Also, the positive control (3.68X10^6^ of *E. coli*) shows a higher effect than the OMV treatment. However, the impact pattern of *E. coli* treatment in wildtype and cav-1 KO was similar to the OMV treatment.

**Figure 2:**
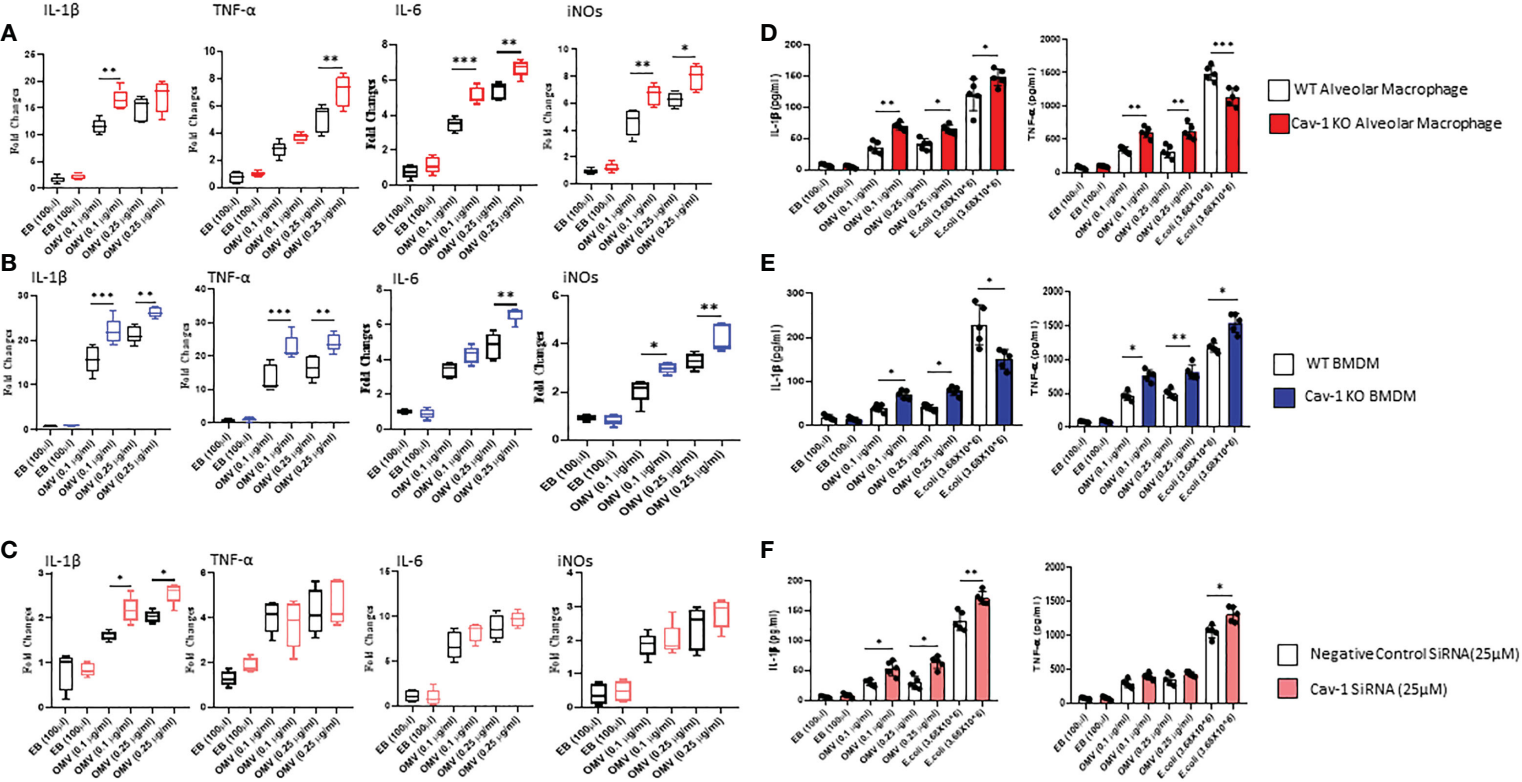
Effect of OMVs on proinflammatory mediator secretion. WT and Cav-1 KO AMФ, BMDM, and human THP-1 cells were treated with different concentrations (0.1 and 0.25 μg/ml) of OMVs or E. Coli (3.68X10^6^); after 24h, gene expression of proinflammatory mediators such as IL1β, TNF-α, IL-6, and iNOs **(A–C)** using qRT PCR method. Secretory levels of IL1β and TNFα **(D–F)** were quantified in the culture supernatant using the ELISA technique. Results were expressed as mean ± SD. Statistical Analysis was performed non-parametrically using the One-way Analysis of variance (ANOVA) with Tukey’s multiple comparison tests to determine significant differences between the experimental groups. *p < 0.05, **p<0.01 and ***p<0.005 set as Statistical significance.

Further, to identify the influence of OMV on MФ activation and release of proinflammatory cytokines, we analyzed gene expression and secretory levels of TNF-α and IL-1β. As shown in **Figures 2A–C**, OMV-treated MФ expressed significantly higher gene expression of pro-inflammatory markers such as IL1-β, TNF-α, IL6, and iNOs than the EB-treated groups. Compared to the wild type MФ, Cav-1-KO significantly induced the gene expression of these proinflammatory mediators. In addition, we also analyzed the secretory level of TNF-α and IL-1β. Results demonstrated that OMV-treated MФ significantly releases higher cytokine levels in the supernatant than EB-treated MФ as determined by ELISA. Interestingly, the deletion of cav-1 exacerbated the effects of OMVs on promoting IL-1β release. This observation was consistent in the OMV-treated cells among all three cell types (**Figures 2D–F**). In alveolar MФ and BMDMs (mouse cells), the deletion of cav-1 also boosted the TNF-α secretion in these primary mouse macrophages (**Figures 2D, E**). However, it was not very significant in human THP-1 (**Figure 2F**). Moreover, we found that deletion of cav-1 had a differential effect on IL-1β and TNF-α secretion from macrophages treated with *E. Coli* (**Figures 2A–F**).

### OMV treatment upregulated the expression of a variety of TLRs. Deletion of cav-1 augmented the effects of OMVs on TLR expression

**Figure 3:**
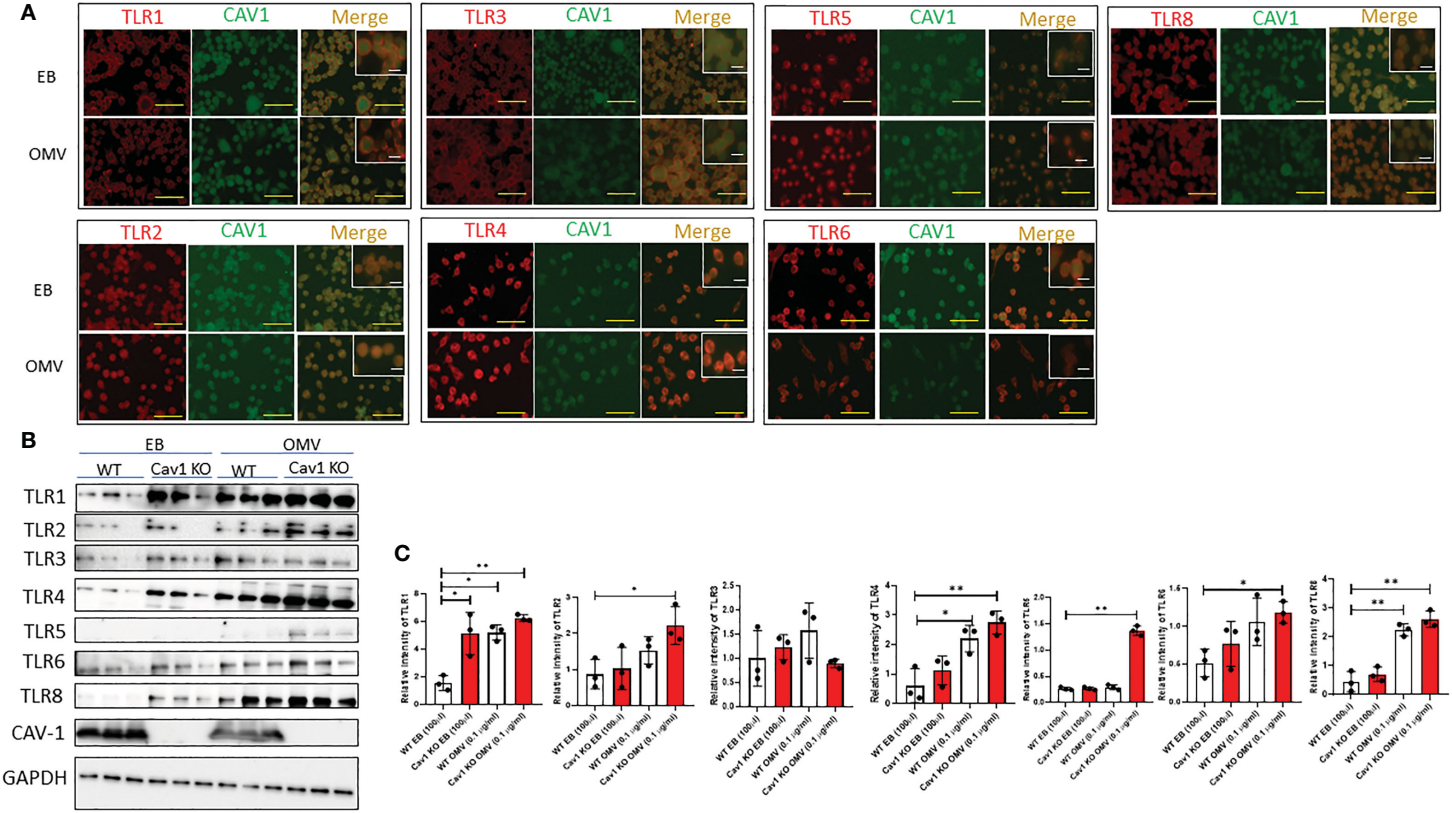
Effect of Cav-1 on the expression of various TLRs induced by OMV. WT AM was treated with 0.1mg/ml of OMV for 3h. Representative fluorescence microscopic images **(A)** demonstrated the expression of different TLRs (red) and cav-1 (green) after EB/OMV treatments. (magnification = ×20; scale bar (yellow) = 50 μm & magnification = ×63; scale bar (white) = 10 μm). Western blot analysis **(B)** demonstrated the expression of different TLRs. Bar graphs **(C)** show the quantification results of TLRs in AM GAPDH was used as internal control, and the control value was considered one unit (n=3). Results were expressed as mean ± SD. Statistical Analysis was performed non-parametrically using the One-way Analysis of variance (ANOVA) with Tukey’s multiple comparison tests to determine significant differences between the experimental groups. *p < 0.05 and **p < 0.01 set as Statistical significance.

TLRs play essential roles in MФ activation in response to various stimuli, including bacteria, viruses, and fungi (36). We first screened whether OMVs modulate the expression of different TLRs in macrophages. The microscopic images revealed that the treatment of OMV markedly reduced the expression of Cav-1 and increased a significant amount of TLRs 3, 4, and 5. There are no significant changes in other TLRs, such as TLRs 1,2,6, and 8 (**Figure 3A** & **Supplementary Figure 1C**). Also, we confirmed the TLRs and Cav-1 expression by the immunoblotting method. In the WT mouse alveolar macrophages, no significant changes were found among TLR2, TLR3, TLR5, and TLR6. The cav-1 expression also is markedly decreased by the OMV treatment (**Figures 3B, C**). Interestingly, the deletion of cav-1 promoted TLR1, TLR2, TLR4, TLR5, TLR6 and TLR8 expression in the OMV-treated groups (**Figures 3B, C**). These results demonstrated that Cav-1 reduction increased the expression of all the TLRs.

### The interaction of Cav-1 and TLR4 has a significant role in OMV-induced TLR4 expression

**Figure 4:**
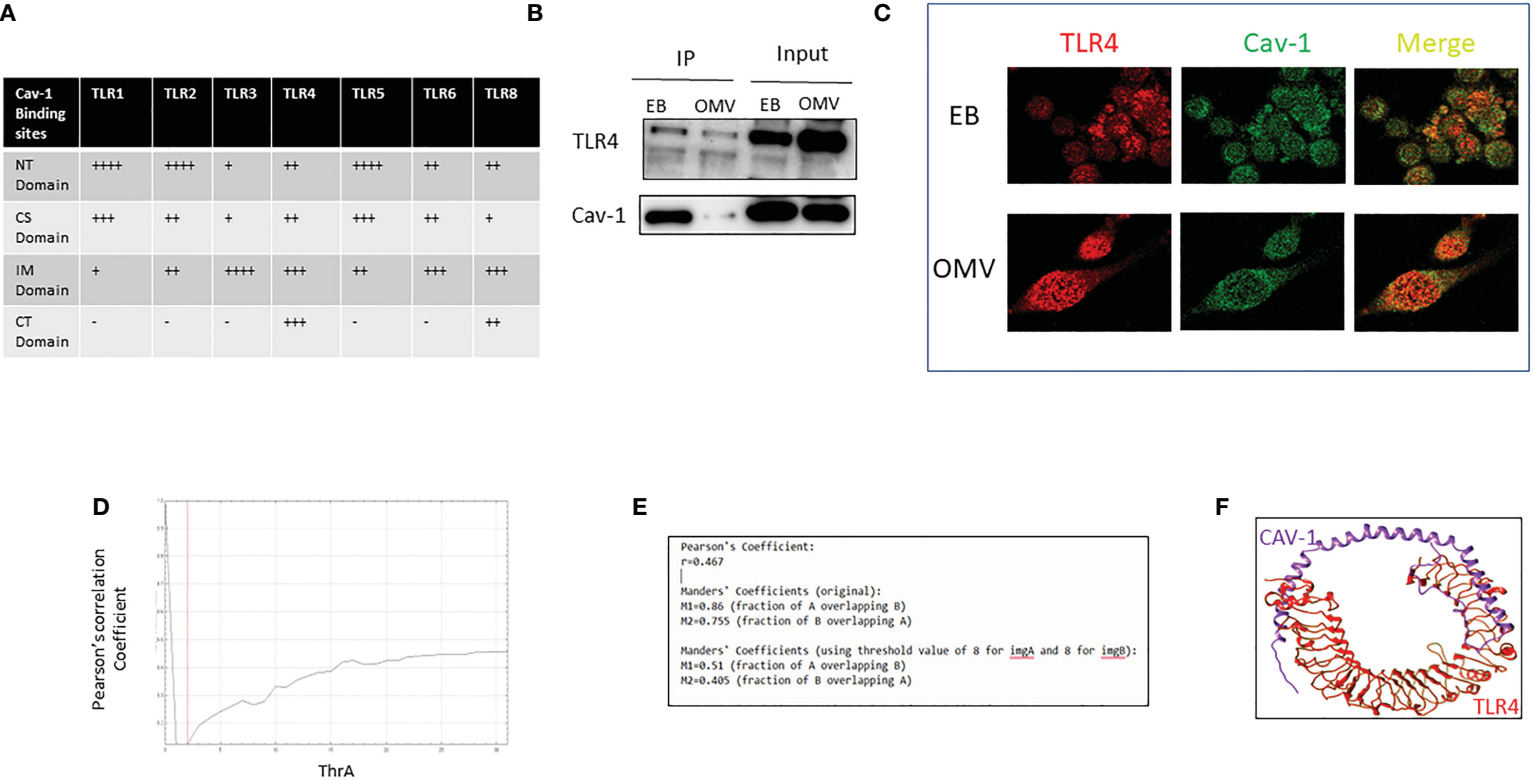
Analysis of the interaction of Cav-1 and TLR4. The table indicates the binding sites of Cav-1 in various TLRs **(A)**. WT AM was treated with 0.1g/ml of OMV for 3h; cell lysate was collected, Cav-1 immunoprecipitated, and TLR4 was detected by the immunoblotting method. 15% of cell lysate was used as input **(B)**. Confocal microscopic images of colocalized TLRs (red) and cav-1 (green) after EB/OMV treatments (magnification = ×63) **(C)**. Graphical representation of values of Pearson's coefficients **(D)**, Colocalization coefficient values using confocal images of TLRs (red) and cav-1 (green) after EB/OMV treatment **(E)**. 3-D image shows the interaction of Cav-1 in the active sites of TLR4 **(F)**. + indicates the binding sites cav-1, - indicates no binding sites of cav-1.

We found that cav-1 regulates all TLRs expression and has been reported to interact physically with all TLRs; Next, we predicted the interactions between cav-1 and TLRs using computational modeling. **Figure 4A** show that differential binding sites on cav-1 were identified using different TLRs. For TLR1, 2, 5, major binding sites located on the N-terminal regions, i.e., NTD domains. On the other hand, for TLR 3, TLR6, and TLR8, the primary binding sites fell into the IMD domains. Compared to other TLRs, it has even binding sites in all the domains in TLR4 (**Figure 4A**). Therefore, further, we confirmed the interaction between Cav-1 and TLR4 by immunoprecipitation, colocalization, and computational models.

In the immunoprecipitation method, lysate of alveolar macrophages treated with EB or OMV was immunoprecipitated with Cav-1 specific antibody, and the immunoblotting analysis determined the level of TLR4. In EB treatment, a higher amount of Cav-1 precipitation coprecipitates a higher amount of TLR4. However, after OMV treatment, cav-1 expression reduced, and a subsequent reduction in TLR4 precipitation (**Figure 4B**). Additional confocal microscopy analysis further showed clusters of TLR4-Alexa Flour 594 and Cav-1-FITC (**Figure 4C**). Colocalization correlation coefficients were measured using confocal microscopic images. Pearson’s coefficient and overlapping values (**Figures 4D, E**), suggested that TLR4 and Cav1 have a moderate positive linear association.

Next, we predicted the interactions between cav-1 and TLR4 using computational modeling. **Figure 4F** shows that differential binding sites on cav-1 were identified with TLR4. Total of 28 residues of cav-1 made bonding with 21 residues of TLR4. Specifically, most of the bonding is hydrogen bonding or non-bonded contacts (**Supplementary Figure 1D**). The predicted binding sites on cav-1 were evenly distributed among all four TLR4 domains. All these results confirm that TLR4 physically interacts and is regulated by Cav-1.

## Discussion

Severe bacterial infection-induced sepsis and multiple organ failure (MOF) often result in an imbalance between host bactericidal effects and excess inflammatory responses (37). Mechanisms underlying this runaway inflammation remain incompletely understood, thus impeding the development of novel therapeutics. Serious concerns exist about Gram-negative (G-) bacteria-induced sepsis, such as a significant propensity to acquire antibiotic treatment resistance (38) and a significantly higher incidence of bacteremia among adult patients with septic shock and SIRS (39). Upon activation, macrophages release early response cytokines/chemokines, which mediate the recruitment of neutrophils, and exudate MФs and lymphocytes to the site of infection, ultimately resulting in the clearance of pathogens (2).

Bacterial outer membrane vesicles (OMVs) are released from the outer membranes of G- bacteria. A recent report showed that wild-type (WT) OMVs trigger inflammatory lung responses and injury *via* TLR4 and TLR2 signaling (17). However, our data suggest that OMVs modulated TLRs 2 and 4 and other TLRs (**Figure 3**).

The first innovative aspect of this study is that we demonstrated that OMVs modulated multiple TLRs, including TLR 1, 2,4, 5, 6, and 8. Our results are consistent with previous reports showing that OMVs contain not only LPS but also phospholipids, peptidoglycan, outer membrane proteins (OMPs), cell wall components (G+ OMVs), periplasmic and cytoplasmic proteins, nucleic acids, and ion metabolites. OMVs induced the TLR 1, 2, 4, 5, 6, and 8 expressions in macrophages, suggesting that each component of OMVs exerted certain effects on promoting macrophage M1 polarization. OMVs may function as a packet full of PAMPs, which trigger multiple signaling pathways simultaneously. In the case of TLR3, it does not get affected by OMVs. TLR3 recognizes double-stranded RNA (dsRNA) (6, 7, 11), so, unsurprisingly, TLR3 was not affected by G-bacteria-derived OMVs. In our report, the deletion of cav-1, the phagocytosis of OMVs was not dramatically promoted, suggesting the participation of cav-1 in regulating OMV’s effects on macrophage M1 activation. Consistently, deletion of cav-1 upregulated TNF-α and IL-1β secretions, as well as ROS generation from macrophages. Interestingly, the deletion of cav-1 upregulated the TLR1 and TLR 8 at the basal level. But it does not impact the cytokine secretions. In the presence of OMVs, the deletion of cav-1 augmented the expression of TLR1, 2,4,5,6, and 8. Among all these TLRs, the deletion of cav-1 and the presence of OMVs were required to upregulate TLR2 and TLR5 expression, suggesting that TLR2 and TLR5 are both involved in OMV signaling in the presence of cav-1. Previous reports have illustrated that TLR2 and TLR4 mediate the signaling pathways from the OMVs derived from actinobacteria (40). TLR5 has also been reported to be physically associated with TLR4 and biases TLR4 signaling towards the MyD88 pathway (7, 11). Therefore, cav-1-regulated TLR 2, 4, and 5 may synergistically play an essential role in OMV-mediated MФ activation. Therefore, the confirmation of interaction between TLR4 and Cav-1 is an essential part of the research on finding the relationship between TLRs and Cav-1. However, Immunoprecipitation, colocalization, and computational modeling show that cav-1 interacts with TLR4, which confirms the previous report (29).

Cav-1 has been identified in various immune cells, including monocytes/macrophages, polymorphonuclear cells (PMNs), mast cells, and lymphocytes. Previously, cav-1 has been implicated as a modulator of innate immunity and inflammation and inhibits the expression of pro-inflammatory cytokines from macrophages by regulating the activation of mitogen-activated protein kinase (MAPK) family members (22). This trans-membrane scaffolding protein usually forms an oligomer and provides a platform to modulate endo/exocytosis and cellular signaling. Cav-1 carries four significant domains. Among them, the scaffolding domain (82–101 amino acids) (CSD) occurs in cav-1 and binds caveolin-binding motifs (CBM). CBMs are present in several proteins, including eNOS, PKA, G-protein, and EGFR, which include conserved motifs rich in aromatic residues. The cav-1 binding proteins include aromatic-rich motifs with the sequences ΦXΦ XXXXΦ, ΦXXXXΦXXΦ, and ΦXΦXXXXΦXXΦ (Φ = aromatic residue, X = any amino acid). Aggregation of cav-1 occurs at residues 94–101 on the C-terminal side of the CSD (VTKYWGYR) of cav-1. Additionally, cav-1 residues 84–94 may form a -sheet hairpin necessary for cav-1 self-oligomerization (27). The N-terminal domain (NTD) denotes the soluble stretch formed by cav-1 residues 1–81. Several critical phosphorylation sites play vital roles in regulating protein-protein interactions within the NTD. At physiological pH, the secondary structure of the cav-1 NTD is mostly disordered and composed of random coils. Our report predicts the interaction between cav-1 and potential binding sites of TLR4 on cav-1. Differential reports exist regarding the role of cav-1 on phagocytosis (41). Others reported that deletion of cav-1 impaired the phagocytosis of macrophages (23). Unfortunately, OMV doesn’t induce phagocytosis in this study. Additional assays should be performed among all the components of OMVs to determine the most important one and whether OMVs exert their function *via* surface antigens or endocytosis by macrophages. The role of cav-1 in OMV endocytosis by macrophages and its function in the activation of other TLRs requires further investigation.

Our brief report showed that OMVs promote macrophage M1 activation *via* multiple TLRs. Cav-1 forms a platform to gather all TLRs by regulating them. However, Cav-1 directly binds with TLR4, and it regulates OMV-induced macrophage polarization.

## Data availability statement

The raw data supporting the conclusions of this article will be made available by the authors, without undue reservation.

## Ethics statement

All animal-related procedures were approved and followed the guidelines by Boston University’s institutional animal care and use committee (IACUC) (# PROTO201800354/PROTO201800355).

## Author contributions

YJ: Conceptualization, experimental design, draft preparation, and supervision. AS: Experimental design, Data curation, and draft preparation. WA and SM: Data curation. HL and BG: Supervision. All authors contributed to the article and approved the submitted version.

## Supplementary material

Supplementary Figure 1
**(A)** Graphical representation of gene expression of Cav-1 after transfection of different concentrations (5,10, 25, and 50 µM) of cav-1 siRNA to THP-1 cells. **(B)** Immunoblotting image of transfection of different concentrations (5,10, 25, and 50 µM) of cav-1 siRNA. **(C)** Bar Graph of Mean fluorescence intensity of the various TLRs (Red bars) and Cav-1 (green bars). **(D)** Amino acid residues and their interaction between Cav-1 (purple) and TLR4 (Red). **(E)** Representative images of cell migration assay under various conditions under a light microscope. Results were expressed as mean ± SD. Statistical Analysis was performed non-parametrically using the One-way Analysis of variance (ANOVA) with Tukey’s multiple comparison tests to determine significant differences between the experimental groups. *p<0.05, **p<0.01 and ***p<0.005 set as Statistical significance.
