# Supplementary figures and images for: Caveolin-1 regulates OMV-induced macrophage pro-inflammatory activation and multiple Toll-like receptors
Source: Front Immunol. 2023 Feb 2;14:1044834. doi: 10.3389/fimmu.2023.1044834 (PMC9933776; doi:10.3389/fimmu.2023.1044834)

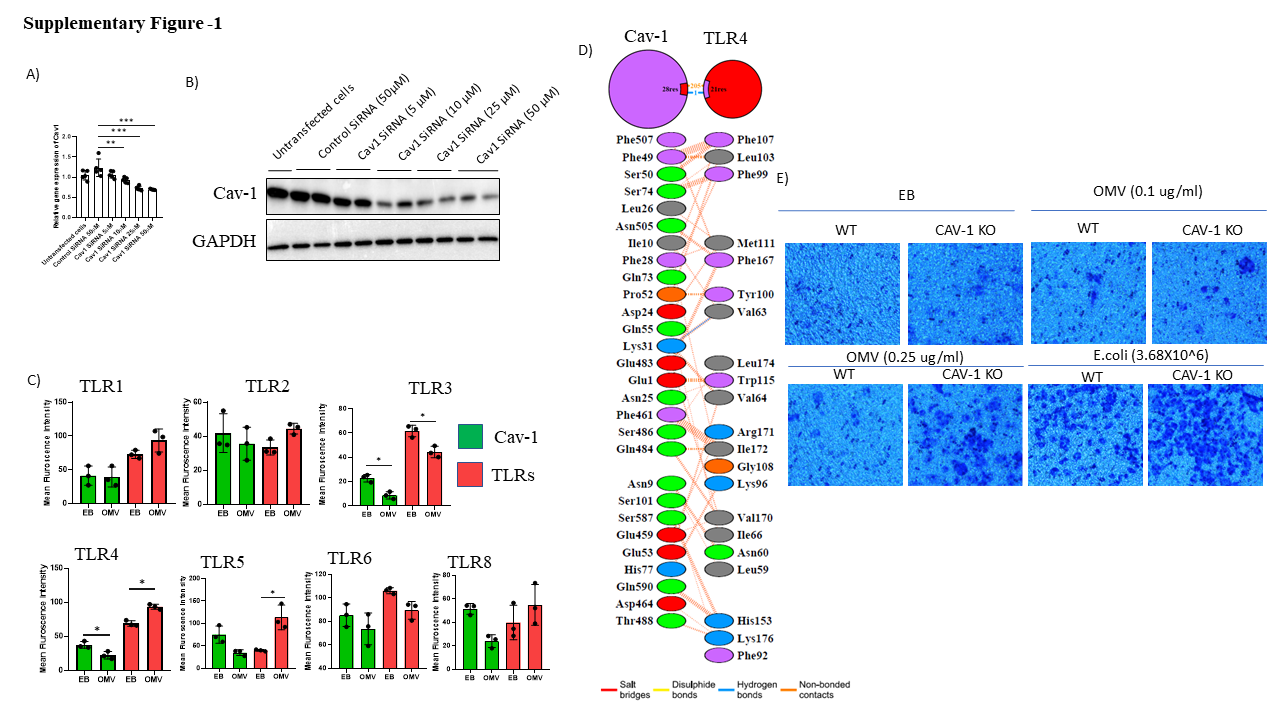

Supplement: Supplementary Figure 1 — (A) Graphical representation of gene expression of Cav-1 after transfection of different concentrations (5,10, 25, and 50 µM) of cav-1 siRNA to THP-1 cells. (B) Immunoblotting image of transfection of different concentrations (5,10, 25, and 50 µM) of cav-1 siRNA. (C) Bar Graph of Mean fluorescence intensity of the various TLRs (Red bars) and Cav-1 (green bars). (D) Amino acid residues and their interaction between Cav-1 (purple) and TLR4 (Red). (E) Representative images of cell migration assay under various conditions under a light microscope. Results were expressed as mean ± SD. Statistical Analysis was performed non-parametrically using the One-way Analysis of variance (ANOVA) with Tukey’s multiple comparison tests to determine significant differences between the experimental groups. *p<0.05, **p<0.01 and ***p<0.005 set as Statistical significance. [file Image_1.tif]
