# Supplementary material for: Caveolin-1 regulates OMV-induced macrophage pro-inflammatory activation and multiple Toll-like receptors
Source: Front Immunol. 2023 Feb 2;14:1044834. doi: 10.3389/fimmu.2023.1044834 (PMC9933776; doi:10.3389/fimmu.2023.1044834)
Supplement: Supplementary file 2 [file Table_1.docx]

Supplemental Table : 1: List of primers used.

| S.No | Primer Name | Primer Sequence |
| --- | --- | --- |
| 1 | TNFα-F | 5′-GACGTGGAACTGGCAGAAGAG-3′ |
|  | TNFα-R | 5′-TTGGTGGTTTGTGAGTGTGAG-3′ |
| 2 | IL1β-F | 5′-GCAACTGTTCCTGAACTCAACT-3′ |
|  | IL1β-R | 5′-ATCTTTTGGGGTCCGTCAACT-3′ |
| 3 | iNOS-F | 5’-CAAGCTGAACTTGAGCGAGGA-3' |
|  | iNOS-R | 5’-TTTACTCAGTGCCAGAAGCTGGA-3' |
| 4 | IL6-F | 5’-GAGGATACCACTCCCAACAGACC-3’ |
|  | IL6-R | 5’-AAGTGCATCATCGTTGTTCATACA-3’ |
| 5 | GAPDH-F | 5’-TGCACCACCAACTGCTTAG-3’ |
|  | GAPDH-R | 5’-GGATGCAGGGATGATGTTC-3’ |
